# Cytotoxic Effects of Newly Synthesized Palladium(II) Complexes of Diethyldithiocarbamate on Gastrointestinal Cancer Cell Lines

**DOI:** 10.1155/2014/813457

**Published:** 2014-07-24

**Authors:** Shahram Hadizadeh, Nowruz Najafzadeh, Mohammad Mazani, Mojtaba Amani, Hassan Mansouri-Torshizi, Ali Niapour

**Affiliations:** ^1^Department of Biochemistry, School of Medicine, Ardabil University of Medical Sciences, Ardabil 5618985991, Iran; ^2^Laboratory of Embryology and Stem Cells, Department of Anatomy and Pathology, School of Medicine, Ardabil University of Medical Sciences, Ardabil 5618985991, Iran; ^3^Department of Biophysics, School of Medicine, Ardabil University of Medical Sciences, Ardabil 5618985991, Iran; ^4^Department of Chemistry, Faculty of Basic Sciences, University of Sistan and Baluchistan, Zahedan 9816745345, Iran

## Abstract

As a part of a drug development program to discover novel therapeutic and more effective palladium (Pd) based anticancer drugs, a series of water-soluble Pd complexes have been synthesized by interaction between [Pd (phen)(H_2_O)_2_(NO_3_)_2_] and alkylenebisdithiocarbamate(al-bis-dtc) disodium salts. This study was undertaken to examine the possible cytotoxic effect of three novel complexes (0.125–64 *µ*g/mL) on human gastric carcinoma (AGS), esophageal squamous cell carcinoma (Kyse-30), and hepatocellular carcinoma (HepG2) cell lines. The cytotoxicity was examined using cell proliferation and acridine orange/ethidium bromide (AO/EB) assay. In order to examine the effects of new Pd(II) complexes on cell cycle status, we performed cell cycle analysis. The complexes were found to have completely lethal effects on the cell lines, and the half maximal inhibitory concentration (IC50) values obtained for the cell lines were much lower in comparison with cisplatin. We demonstrated that the three new Pd(II) complexes are able to induce G2/M phase arrest in AGS and HepG2; in addition, the Pd(II) complexes caused an S phase arrest in Kyse-30 cell line. Our results indicate that newly synthesized Pd(II) complexes may provide a novel class of chemopreventive compounds for anticancer therapy.

## 1. Introduction

Cancer is a major public health problem in the world and more patients are afflicted with gastrointestinal cancer [1]. According to GLOBOCAN 2008 [2], there are increased incidence rates of the esophageal [3], gastric [4, 5], colon [6, 7], and hepatic [8, 9] cancers. In the world, about 12.7 million cancer new cases and 7.6 million cancer deaths are estimated to have occurred [2]. A substantial proportion of the cancer morbidity could be prevented through early detection and treatments, which renders the quest for highly effective antineoplastic agents [6].

Cisplatin is one of the most successful anticancer compounds that have been used clinically for more than 40 years. It is commonly used to treat several human cancers such as testicular [10] and ovarian [11] cancers and also it is widely employed for treating bladder [12], cervical [13], head and neck [14], esophageal [15], and nonsmall cell lung [16] cancers.

The biological activity of cisplatin cytotoxicity involves the binding of the drug to DNA and non-DNA targets and inhibits transcription and DNA replication that finally leads to apoptosis, necrosis, or both of them. The main site of cisplatin attack to DNA is N7 atoms of guanine and adenine located in the major groove of the double helix [17]. Furthermore, DNA is not the only target for platinum(Pt) complexes, but many other cell components, including glutathione, methionine, and other S-containing biomolecules, can play this role [18–20]. Cisplatin resistance is a mechanism that includes decreased drug accumulation in cancer cells, inactivation by thiol containing biomolecules (glutathione), and enhanced repair of DNA damage [21–23].

Therapeutic use of cisplatin is limited due to its side effects such as ototoxicity, nausea, nephrotoxicity, and gastrointestinal and bone marrow toxicity as well as resistance to this drug. Recently, second-generation cisplatin analogues such as oxaliplatin, satraplatin (JM216), and LA-12 are described to have hepatotoxicity and nephrotoxicity milder than cisplatin [24]. Most anticancer compounds are ineffective in the treatment of malignant cancers; therefore, much attention has been focused to find more effective and less toxic complexes than the existing pharmaceuticals. Thus, attention has turned to other Pt group elements (Pd, ruthenium, iridium, rhodium, and osmium) and novel design strategy of metal complexes containing N and S donor ligands such as sodium diethyldithiocarbamate [25–29].

The Pd complexes display moderate activities; until now, none has yet been proven to be effective in patients. Recently, some new Pd(II) and P(II) complexes have been synthesized to combine anticancer activity and reduce toxic effects of cisplatin [30]. More recently, we have been studying some diimine Pt(II) and Pd(II) complexes of dithiocarbamate derivative as potential anticancer agents [31].

In the present work, three novel Pd(II) complexes with a dithiocarbamate(dtc) with the formulas (complex 1 = *μ* − 1, 3-propylene bis(dithiocarbamate) bis(1,10-phenanthroline Pd(II)) nitrate), (complex 2 = *μ* − 1, 4-butylene bis(dithiocarbamate) bis(1,10-phenanthroline Pd(II)) nitrate), and (complex 3 = *μ* − 1, 8-octylene bis(dithiocarbamate) bis(1,10-phenanthroline Pd(II)) nitrate) have been synthesized and characterized. Previously, three Pd(II) complexes of M(2,2′-bipyridine) (morpholine dithiocarbamate) NO_3_ Pd(II) complexes, 1,10-phenanthroline hexyl dithiocarbamatopalladium (II) nitrate [32], and *α*-diimine platinum(II) and Pd(II) dithiocarbamate complexes [31] have been synthesized and characterized by good anticancer activity. However, dithiocarbamate complexes of metals could be more interesting and they display cytotoxic properties [30]. Thus, in this study, we report the cytotoxicity of three palladium(II) complexes with dithiocarbamate ligands and the effect of hydrocarbon chain lengths (propylene, butylene, and octylene) in the structure of these complexes on the anticancer activity compared to cisplatin. The Pd(II) complexes are expected to bind DNA by intercalation [33], a mechanism different from that of interaction of cisplatin with DNA. Metal complexes that contain 1,10-phenanthroline have been known to have effective anticancer activity and also to be intercalated between the DNA base pairs [34–36].

Therefore, since several reports have represented the fact that Pd(II) complexes containing dithiol group have low side effects especially on the kidney [30] and based on the chemopreventive cytotoxicity of the three novel Pd(II) complexes and also chemosensitivity of esophageal, gastric, and liver cancers, we have decided to investigate the cytotoxicity of these complexes against gastrointestinal cancer cell lines of AGS, KYSE-30, and HepG2.

## 2. Material and Methods

### 2.1. Culture of Cell Lines

The cell lines used in this study included AGS, HepG2, and KYSE-30. The cell lines were obtained from the National Cell Bank of Iran (NCBI, Pasteur Institute of Iran, Tehran). The cells were maintained in RPMI 1640 (Cat. number 51800-035, Gibco, UK) medium supplemented with 10% heat-inactivated fetal bovine serum (FBS; Cat. number 10270-106, Gibco, UK), 2 mM L-glutamine, penicillin, and streptomycin. The cells were incubated at 37°C in a humidified atmosphere with 5% CO_2_ until they reached 70–80% confluence. Then, the cells were detached by 0.25 (w/v) trypsin and 0.02 (w/v) ethylenediaminetetraacetic acid (EDTA) and were then plated in a 96-well for cytotoxicity assessment.

### 2.2. Chemicals

A water soluble series of newly synthesized Pd(II) complexes of formula [(phen) Pd (*μ*-al-bis-dtc) Pd(phen)](NO_3_)_2_ (where alkylenebisdithiocarbamate, al-bis-dtc = propylenebisdithiocarbamate (pn-bis-dtc,** 1**); butylenebisdithiocarbamate (bu-bis-dtc,** 2**); and octylenebisdithiocarbamate (oc-bis-dtc,** 3**) and phen = 1,10-phenanthroline) have been synthesized similar to the procedure outlined by Islami-Moghaddam et al. [25], by interaction between [Pd(phen)(H_2_O)_2_](NO_3_)_2_] and alkylenebisdithiocarbamate(al-bis-dtc)disodium salts. In these binuclear Pd(II) complexes, 1,10-phenanthroline (phen) acts as capping ligand and al-bis-dtc bridges the two Pd centers. Cisplatin was purchased from Merck (232120) and dissolved in dimethyl sulfoxide and added to the medium at 0.125–32 *μ*g/mL concentrations

### 2.3. Cell Proliferation Assay

The effects of the Pd(II) complexes on cell viability were determined using 3-(4,5-dimethylthiazol-2-yl)-2,5-diphenyltetrazolium bromide (MTT) assay [37]. The AGS, Kyse-30, and HepG2 cells were plated at a density of 1 × 10^4^ cells per well in 200 *μ*L medium and incubated overnight; next, the medium was exchanged with 0.125–64 *μ*g/mL of three new Pd(II) complexes and indicated concentration of cisplatin in FBS-free RPMI 1640 medium. Afterwards, plates were incubated for 24 and 48 hrs. Then, the supernatant was removed and the MTT solution (5 mg/mL in PBS, 20 *μ*L, Sigma, M2128) was added to each well. Plates were incubated again for additional 4 hrs at 37°C in a 5% CO_2_. The supernatants were removed and DMSO (200 *μ*L) was added to each well. Plates were shaken for 10 min to dissolve the precipitate. The sample absorbance was read at 540 nm using a BioTek Synergy HT microplate reader (BioTek Instruments Inc., USA).

### 2.4. AO/EB Staining

AGS, Kyse-30, and HepG2 cell lines were seeded in six-well plates at a density of 1 × 10^3^ cells per well and incubated overnight and then various concentrations (0.125–64 *μ*g/mL) of the new Pd(II) complexes were added to each well and were incubated for 72 hrs. After the incubation, the plates were centrifuged for 5 min (129 g, 1,000 rpm) at 4°C and were washed with phosphate buffer saline (PBS). The EB/AO dye mix (100 *μ*g/mL of AO and 100 *μ*g/mL of EB) was prepared in PBS and 100 *μ*L was added to each well. Cells were viewed under an inverted fluorescence microscope (IX 71, OLYMPUS) and pictures were taken with a digital camera (DP 71, OLYMPUS). Tests were conducted in triplicate by counting a minimum of 100 total cells each by ImageJ software. Live cells were determined by the uptake of AO (green fluorescence). EB is only able to pass through the ruptured membrane of late apoptotic and necrotic cells. Live and dead apoptotic cells were identified by perinuclear condensation of chromatin stained by AO or EB, respectively, and by the formation of the apoptotic bodies. Necrotic cells were identified by uniform labeling of the cells with EB (red fluorescence) [38–40].

### 2.5. Cell Cycle Analysis

Cell cycle phase alterations were determined by analytical DNA flow cytometry. Briefly, the cells were seeded in 25 cm^2^ flasks at a density of 1 × 10^6^ cells. The cells were incubated for 24 hrs with IC50 of the Pd(II) complexes and control was maintained in RPMI-1640 medium supplemented with 10% FBS. After the treatment, the DNA content and cell cycle distribution were determined by flow cytometry. The cells were washed with cold PBS twice and fixed in 70% ethanol at 4°C at least 4 hrs. The fixed cells were centrifuged (300 g, 4°C, 5 min) and washed with cold PBS and then stained with 4,6-diamidino-2-phenylindole dihydrochloride (DAPi, 1 *μ*g/mL, Triton X-100 0.1% v/v in PBS) for 30 min at 37°C in the dark. The stained cells were then transferred to flow tubes by passing through a 30 *μ*m nylon mesh filters. Flow cytometric analysis was performed using a flow cytometer (Partec CyFlow Space, Germany). The distribution of cells in different cell cycle phases was analyzed using Partec FloMax software [41].

### 2.6. Statistical Analysis

Statistical analyses were performed using SPSS 16.0 software. Statistical differences among treated and control cells were determined by one-way ANOVA (analysis of variance) followed by Tukey post hoc comparison test and mean differences with *P* < 0.05 were considered statistically significant. Dose response curves and IC50 values were generated using Sigma Plot10 (Systat Software, CA).

## 3. Results

### 3.1. Growth Inhibition Study

The anticancer effects of the Pd(II) complexes against AGS, HepG2, and KEYSE-30 cancer cell lines were assessed by MTT assay. Cell viability was determined using the MTT assay after treatment with the Pd(II) complexes for 24 and 48 hrs. It was found that the complexes exhibited cytotoxic effects in a dose dependent manner. According to the dose response curves, the complexes had strong growth inhibitory effects on AGS, Kyse-30, and HepG2 cells.

Furthermore, the IC50 values of the complexes were compared to cisplatin (Table 1). These values for Pd(II) complexes are much lower as compared to those achieved for cisplatin reported in this paper. The antitumor activity varied depending on the cell line type and concentration of the complexes. The difference between antitumor activities of the Pd(II) complexes is noticed and, in general, the activities were the same. The analysis of IC50 values showed that Pd(II) complexes 1 and 2 were more cytotoxic against AGS, Kyse-30, and HepG2 cells than complex 3. The best cytotoxic effects were achieved by the Pd(II) complex 1 (IC50 = 0.68) on AGS cells (Table 1 and Figure 3).

### 3.2. AO/EB Staining for Apoptotic Cells

Morphological characteristics of the new Pd(II) complexes induced cell death were determined by AO/EB staining shown in Figure 2. The results showed that, after incubation at 0.125–64 *μ*g/mL of the three new Pd(II) complexes for 72 hrs, a series of morphological changes, including condensation and fragmentation of chromatin and nucleus and formation of apoptotic bodies, was observed which was the evidence of apoptosis. In contrast to the treated cells, viable cells exhibited normal and green nuclei appearances. After the incubation of AGS, HepG2, and KEYSE-30 cell lines with the tested compound, the decrease of cell viability in all experimental series was observed. It was correlated with the increase of concentration of the Pd(II) complexes.

### 3.3. Effects of the Pd Complexes on the Cell Cycle

Flow cytometric analysis demonstrated the growth inhibitory effects of the complexes on cell cycle progression. The cancer cell lines were treated with increasing concentrations of the Pd(II) complexes in complete medium and the effective dose was determined to be at the concentration close to IC50 values to obtain significant cell cycle arrest (Figure 4). The percentages of cells in G0/G1, S, and G2/M phase were calculated using Partec FloMax software and were shown in Table 2 and Figure 4. Flow cytometry analysis demonstrated that, with the IC50 values of the complexes, the population of AGS and HepG2 cells in the G2/M checkpoint was increased significantly with respect to controls. After 24 hrs, only 24.88% of untreated AGS cells were in G2/M phase, but 37.84% of 0.68 *μ*g/mL complex 1, 39.47% of 0.78 *μ*g/mL complex 2, and 35.31% of 0.94 *μ*g/mL complex 3 treated cells and HepG2 (*P* < 0.05 for 36.57%, 35.90%, and 39.18 of complexes-1–3 treated cells with respect to 27.68% of untreated cells) cell lines were in G2/M phase, respectively. The Pd complexes caused an S phase arrest in Kyse-30 cell line (measured at 24 h after treatment), which is not expected since Pd(II) complex treatment leads to DNA damage in the G2-M phase of the cell cycle (*P* < 0.05, for 23.92%, 21.31%, and 25.17% of complexes-1–3 treated cells, with respect to 16.6% of untreated cells, resp.) (Table 2 and Figure 4).

## 4. Discussion

**Figure 1:**
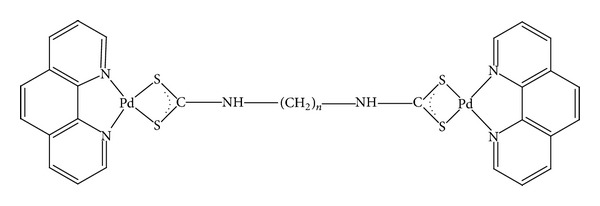
The schematic structure of Pd(II) complexes (complex 1: *n* = 3, complex 2: *n* = 4, and complex 3: *n* = 8).

**Figure 2:**
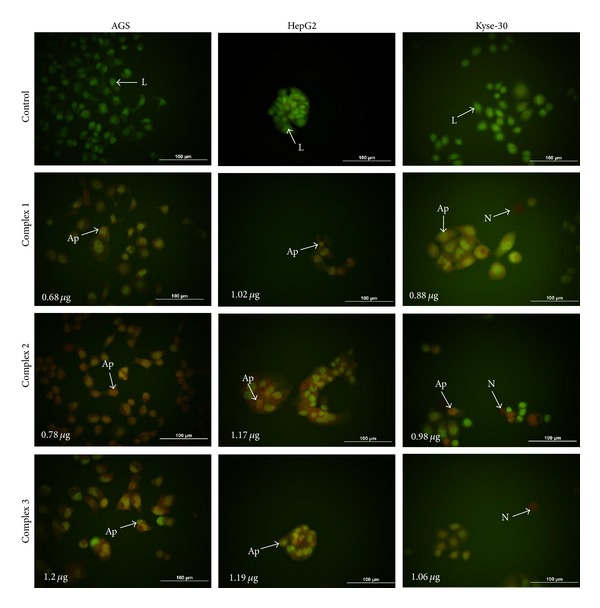
Photomicrographs of AO/EB-stained control AGS, Kyse-30, and HepG2 cells and the cells treated with IC50 of the Pd complexes (1–3) for 72 hrs. Representative photomicrographs show live, apoptotic, and necrotic cells treated with the Pd complexes. L: Live; Ap: apoptotic; N: Necrotic.

**Figure 3:**
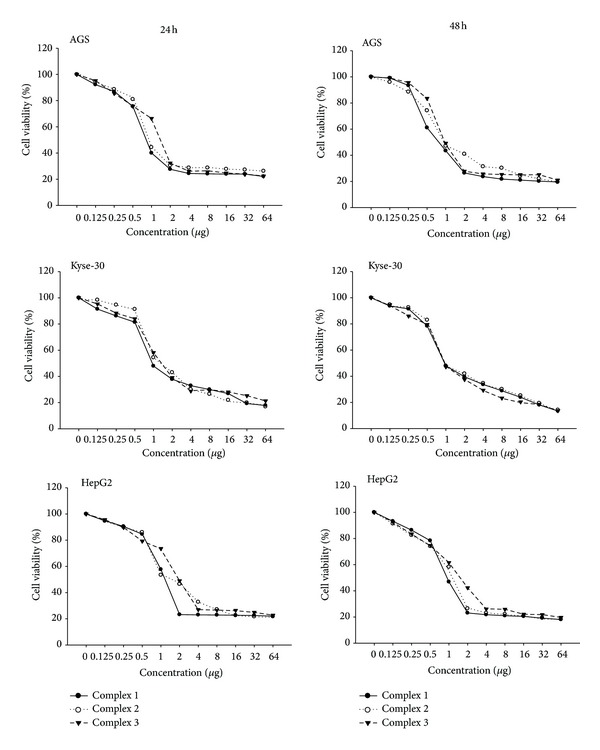
Representative graphs of AGS, Kyse-30, and HepG2 cells survival after 24 and 48 hrs of cell growth in the presence of the three Pd(II) complexes.

**Figure 4:**
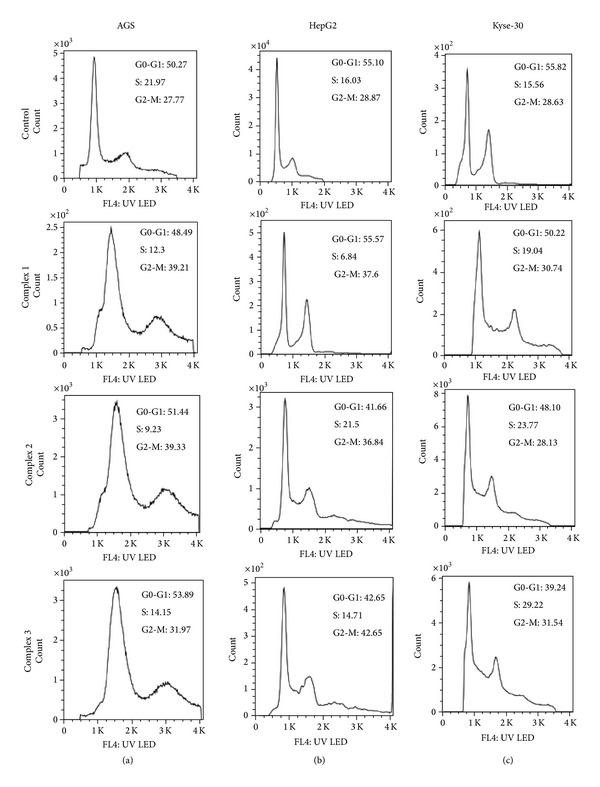
Palladium complex induced cell cycle arrest. AGS, Kyse-30, and HepG2 cell lines were incubated with IC50 of the Pd complexes. After the incubation for 24 hrs, the cells were harvested and stained with DAPi, and DNA content was assessed by flow cytometry. The percentage of cells in each phase was plotted as a function of IC50 of the Pd complexes. The progression of AGS (a) and HepG2 (b) cell cycle was blocked in G2-M when treated with IC50 of three Pd complexes; hence, they stop Kyse-30 cell cycle progression in the S phase (c).

Since the discovery of cisplatin, many new Pt and Pd complexes have been synthesized and evaluated for their possible cytotoxic activity. However, a few of them were recently approved and carboplatin and oxaliplatin are being used as an anticancer drug against several human cancers [21, 42]. Despite the common clinical use of chemotherapeutic agents, cancer recurrence results in the death of many patients due to resistance to chemotherapy [23]. Therefore, there have been many attempts to find complexes which might serve as less toxic and more effective anticancer drugs [43, 44]. In this study, we synthesized three novel Pd(II) complexes. The chemical characteristics of the three novel Pd complexes prompted us to test its potential anticancer activity in vitro. In fact, due to detoxicant properties of dithiocarbamate against heavy metal intoxication, it is possible that diethyldithiocarbamate reduced the toxicity of these three Pd complexes. In the present study, we have investigated the cytotoxic activity and mechanism of action of the three novel Pd(II) complexes on AGS, HepG2, and KYSE-30 cancer cell lines. We demonstrated that the new complexes likely behave in a cytotoxic manner towards the cancer cell lines. On the basis of the MTT it was shown that the three Pd(II) complexes exerted stronger cytotoxic influence on the cell line. In all cell types the IC50 values were lower when compared to cisplatin. All the cell lines were susceptible. Generally, at lower concentrations (0.68–1.2 *μ*g), the Pd(II) complexes induced similar levels of cell death. Therefore, fewer drugs are required to induce cancer cell death and thus can be tolerated by patients. Significantly, this study shows the importance of using a panel of cell lines and Pd(II) complexes. Taking this into account, we can conclude that the cytotoxic activity of three novel Pd(II) complexes may also be related to its geometric structure and size of the molecule (Figure 1).

These data are consistent with those obtained from other studies, which revealed that diimine Pd(II) complexes of alkyldithiocarbamate derivatives had anticancer properties on hepatocellular carcinoma, human ovarian carcinoma, and human lung adenocarcinoma cancer cells [43].

Recently, different complexes of Pd were synthesized and their cytotoxic effects were evaluated by our group. In our previous study, we reported the synthesis and cytotoxicity of two new metal complexes, namely, 2,2′-bipyridinebutyldithiocarbamatoplatinum(II) and Pd(II). We demonstrated that these compounds have strong antitumor activity against chronic myelogenous leukemia cell line, K562, compared to cisplatin [44]. In another study, Mansouri-Torshizi et al. revealed that the *α*-diimine Pt(II) and Pd(II) dithiocarbamate complexes had anticancer properties against K562 and the obtained IC50 values for Pd(II) (IC50 = 0.007 mM,) complexes were lower than those for cisplatin (IC50 = 0.154 mM). Furthermore, the analysis of the interaction of these novel compounds with CT-DNA suggests that they can intercalate in DNA [27]. In another study, Ulukaya et al. used new Pd complex ([PdCl(terpy)] (sac)*·*4H_2_O, (sac = saccharinate, and terpy = 2,2′ : 6′,2′′-terpyridine) against six prostate cancer cell lines, cancer stem cells, and primary culture. They found that the Pd complexes cause DNA damage, cell death, and autophagy [45].

The pattern of cell death was studied by AO/EB. Measurement of AO/EB-stained nuclei showed that the color of the nuclei of cells treated with the three Pd(II) complexes was orange or red. The changes in the color of nuclear chromatin were accompanied by chromatin condensation and fragmentation, which was also observed after Pd(II) treatment. The results demonstrated that the new complexes apply cytotoxic effect via an apoptotic pathway.

The mechanism for the inhibition was further investigated with cell cycle analysis. Following treatment with the Pd complex, the AGS and HepG2 cell lines showed an increase in cells with G2-M DNA content, except in Kyse-30 cells where they appear to be arrested in S phase. Overall, it appears that the Pd complex had different effects on the cell cycle status in various cell lines. In the study of Mukherjee et al. using Pd complexes with a hydrazone ligand, prostate cancer (PC-3) cell growth was inhibited at G2/M phase and apoptosis induced by activation of caspase-3 [46]. Metal complexes containing 1,10-phenanthroline are known to produce DNA adducts by intercalation, which inhibit replication and transcription of DNA [33].

## 5. Conclusion

In conclusion, the new Pd(II) complexes are more efficient in their action on gastric, hepatic, and esophageal cancer cell lines than cisplatin. Importantly, they also successfully inhibited the viability of cancer cells at lower concentrations, implying that these Pd(II) complexes may be used for treatment of cancer. Although we have shown that he novel Pd complexes mediated cell cycle arrest at G2/M and S phases. Finally, the understanding of in vivo effects of three new complexes needs to be investigated further, especially with animal tumor models to confirm its anticancer and chemotherapeutic activity in vivo.

## Figures and Tables

**Table 1:** IC50 (*µ*g)∗ values of the complexes on AGS, Kyse-30, and HepG2 cell lines, as determined by MTT (24 and 48 hrs) assay. The cytotoxic activity of three novel Pd(II) complexes was significantly higher (*P* < 0.05) in comparison with cisplatin.

Cell lines	AGS			Kyse-30			HepG2		
	24 h	48 h	Total IC50	24 h	48 h	Total IC50	24 h	48 h	Total IC50
Complex 1	0.74 ± 0.11	0.63 ± 0.12	**0.68 ± 0.12**	0.87 ± 0.11	0.89 ± 0.25	**0.88 ± 0.18**	1.00 ± 0.01	1.04 ± 0.30	**1.02 ± 0.16**
Complex 2	0.80 ± 0.04	0.75 ± 0.22	**0.78 ± 0.13**	1.02 ± 0.20	0.95 ± 0.31	**0.98 ± 0.26**	1.12 ± 0.10	1.23 ± 0.20	**1.17 ± 0.15**
Complex 3	1.48 ± 0.46	0.94 ± 0.16	**1.2 ± 0.31**	1.05 ± 0.23	1.06 ± 0.22	**1.06 ± 0.23**	1.30 ± 0.15	1.09 ± 0.07	**1.19 ± 0.11**
Cisplatin	4.40 ± 1.56	3.76 ± 0.3	**4.08 ± 0.93**	1.79 ± 0.30	2.00 ± 0.46	**1.9 ± 0.38**	2.07 ± 0.05	2.09 ± 0.15	**2.079 ± 0.10**

*Mean values ± standard deviation from experiments.

**Table 2:** The Pd(II) induced cell cycle arrest. The cell cycle progression blockage was seen in G2-M after 24 hrs of treatment with the IC50 of the Pd(II) complexes.

	AGS			Kyse-30			HepG2		
	G0-G1%	S%	G2M%	G0-G1%	S%	G2M%	G0-G1%	S%	G2M%
Control	53.85 ± 1.95	21.27 ± 3.50	24.88 ± 2.20	54.39 ± 1.32	16.6 ± 1.59	29.01 ± 1.83	55.97 ± 0.4795	16.35 ± 0.99	27.68 ± 1.86
Complex 1	50 ± 1.44	12.15 ± 0.59	37.84 ± 1.66^a^	53.01 ± 3.48	23.92 ± 0.26^a^	22.4 ± 3.16	52.04 ± 3.06	11.39 ± 4.84	36.57 ± 2.76^a^
Complex 2	51.24 ± 4.65	9.29 ± 0.56	39.47 ± 4.1^a^	49.98 ± 1.68	21.31 ± 2.13^a^	28.70 ± 4.98	47.41 ± 4.2	16.68 ± 1.53	35.90 ± 0.97^a^
Complex 3	52.85 ± 3.77	11.83 ± 6.95	35.31 ± 4.16^a^	47.96 ± 7.65	25.17 ± 3.52^a^	26.87 ± 4.13	46.64 ± 4.64	14.19 ± 2.20	39.18 ± 3.15^a^

The data were pooled from three independent tests and were presented as mean ± STDEV; ^a^<0.05.
